# Trans-activation-based risk assessment of *BRCA1* BRCT variants with unknown clinical significance

**DOI:** 10.1186/s40246-018-0183-1

**Published:** 2018-11-20

**Authors:** Jonas Langerud, Elisabeth Jarhelle, Marijke Van Ghelue, Sarah Louise Ariansen, Nina Iversen

**Affiliations:** 1 Department of Medical Genetics, Oslo University Hospital, Oslo, Norway; 2 Department of Medical Genetics, Division of Child and Adolescent Health, University Hospital of North Norway, Tromsø, Norway

**Keywords:** *BRCA1*, HBOC, BRCT, Trans-activation, Functional assay, VUS

## Abstract

**Background:**

Deleterious variants in the tumour suppressor *BRCA1* are known to cause hereditary breast and ovarian cancer syndrome (HBOC). Missense variants in *BRCA1* pose a challenge in clinical care, as their effect on protein functionality often remains unknown. Many of the pathogenic missense variants found in *BRCA1* are located in the *BRCA1* C-terminal (BRCT) domains, domains that are known to be vital for key functions such as homologous recombination repair, protein-protein interactions and trans-activation (TA). We investigated the TA activity of 12 *BRCA1* variants of unknown clinical significance (VUSs) located in the BRCT domains to aid in the classification of these variants.

**Results:**

Twelve *BRCA1* VUSs were investigated using a modified version of the dual luciferase TA activity assay (TA assay) that yielded increased sensitivity and sample throughput. Variants were classified according to American College of Medical Genetics and Genomics (ACMG) criteria using TA assay results and available data. In combining our TA-assay results and available data, in accordance with the ACMG guidelines for variant classification, we proposed the following variant classifications: c.5100A>G, c.5326C>T, c.5348T>C and c.5477A>T as likely benign (class 2) variants. c.5075A>C, c.5116G>A and c.5513T>G were likely pathogenic (class 4), whereas c.5096G>A likely represents a likely pathogenic variant with moderate penetrance. Variants c.5123C>T, c.5125G>A, c.5131A>C and c.5504G>A remained classified as VUSs (class 3).

**Conclusions:**

The modified TA assay provides efficient risk assessment of rare missense variants found in the BRCA1 BRCT-domains. We also report that increased post-transfection incubation time yielded a significant increase in TA assay sensitivity.

**Electronic supplementary material:**

The online version of this article (10.1186/s40246-018-0183-1) contains supplementary material, which is available to authorized users.

## Background

Breast cancer is the most prevalent cancer in women worldwide, representing 25.1% of all new cancer cases and 14.7% of cancer-related deaths [1]. Roughly, 10% of breast cancer incidents can be attributed to pathogenic germline variants. These germline variants are inherited in an autosomal dominant manner and result in what is known as hereditary breast and ovarian cancer syndrome (HBOC) [2]. HBOC confers a 45–65% lifetime risk of developing breast cancer and a 11–44% risk of ovarian cancer in addition to the association with an increased risk of tumour development in other tissues exposed to elevated hormone levels, such as the fallopian tubes, pancreas and prostate [3, 4]. Monoallelic variants in the high penetrance genes *BRCA1* and *BRCA2* are estimated to account for 30% of HBOC cases [2, 5].

The *BRCA1*, *DNA repair associated* (*BRCA1*) gene encodes a 220 kDa nuclear phosphoprotein, consisting of 1863 amino acids (aa). N-terminally, the protein contains a Really Interesting New Gene (RING) domain (aa 8–96) with E3-ubiquitin ligase activity [6]. BRCA1 also includes a nuclear export signal (aa 81–99), a non-canonical NLS (aa 252–257), two canonical nuclear localisation signals (NLS; aa 503–508 and aa 607–617), a coiled-coil domain (aa 1364–1437) and various binding sites and phosphorylation targets for a variety of protein interaction partners [7–10]. C-terminally, BRCA1 contains a domain consisting of two BRCA1 C-terminal (BRCT) domains with trans-activation activity [11, 12]. The BRCT domains are located at aa 1646–1736 and aa 1760–1855 [6].

BRCA1 is directly involved in homologous recombination repair (HRR), and as such is vital for maintaining genomic stability [13]. Deleterious variants in the BRCA1 BRCT domains may halt the interactions between BRCA1 and important facilitators of HRR such as Abraxas, BRCA1 interacting protein C-terminal helicase 1 (BRIP1) or RB-binding protein 8, endonuclease (RBBP8; alias: CtIP) [14]. BRCA1 also regulates the progression of the cell cycle through the S-phase [15, 16] and is associated with the G2/M checkpoint control [17–20]. Additionally, BRCA1 interact with oestrogen receptor-α (ER-α) and is important for the regulation of transcription factors involved in epithelial mesenchymal transition [21–23]. Furthermore, BRCA1 has been implicated in enhancing nucleotide excision repair and transcription-coupled repair via its connection to the RNA polymerase II holoenzyme complex [24]. In addition to the abovementioned roles, BRCA1 has been shown to possess trans-activation (TA) activity, and pathogenic variants in the BRCT domains can abrogate this ability, indicating the importance of TA as a mechanism of tumour suppression [12].

**Fig. 1:**
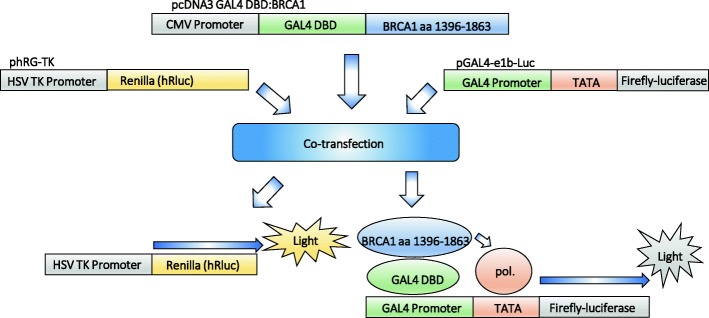
A schematic representation of the trans-activation assay (TA assay). Variant plasmid pcDNA3 GAL4 DBD:BRCA1(aa 1396–1863) is co-transfected with reporter plasmids pGAL4-e1b-Luc and phRG-TK into mammalian cells. Expression of the variant plasmid creates fusion proteins with GAL4 DBD and the BRCA1 BRCT-domains, which bind to the GAL4-specific promoter on the pGAL4-e1b-Luc reporter plasmid and induce expression of Firefly luciferase, in the absence of deleterious variants. The phRG-TK reporter plasmid functions as an internal control

With the rising number of patients undergoing predictive *BRCA1/2* screening, the incidence of variants of unknown clinical significance (VUS) increases. The correct classification of these rare variants is paramount for the right clinical assessment of the patient. Accordingly, in order to classify the 12 *BRCA1* BRCT VUSs found in our patients, we optimised the sensitivity and efficiency of a TA assay (Fig. 1).

## Methods

### Variants included in this study

Twelve missense variants in the *BRCA1* BRCT domains were found in patients during routine diagnostics at the Oslo University Hospital, Department of Medical Genetics. These variants were chosen for functional trans-activation studies (Table 1). Additionally, we tested two variant combinations *in cis* (c.5075A>C/c.5411T>A and c.5252G>A/c.5477A>T) to investigate possible additive or synergistic effects. Variant annotation follows HGVS nomenclature. The reference sequence used was *BRCA1* NM_007294.3 (custom exon numbering). The classifications of the included variants at the beginning of this study were performed at the Oslo University Hospital, Department of Medical Genetics prior to implementation of the ACMG criteria.

**Table 1:** Variant entries in databases dbSNP, ClinVar and HGMD, as well as allele frequencies reported by gnomAD and ESP (ALL: All, AFR: African/African American, NFE: Non-Finnish European, AMR: Latino, EAS: East Asian, SAS: South Asian, OTH: Other)

HGVS nucleotide variant	HGVS protein variant	Exon	Type	dbSNP	ClinVar	gnomAD	ESP	HGMD	SIFT	Align GVGD	Mutation taster	Splice prediction	Class
c.4956G>A	p.(Met1652Ile)	16	Missense	rs1799967	RCV000112434.6: Benign (ENIGMA) RCV000048709.9: Benign/Likely benign RCV000034756.3: Benign RCV000476093.1: Benign RCV000128916.4: Benign/VUS RCV000120261.7: Benign	ALL: 1.82% AFR: 0.18% AMR: 0.40% EAS: 0.012% SAS: 3.78% NFE: 1.53% FIN: 5.08% OTH: 1.66%	EA: 1.5%AA: 0.2%	CM014325(Disease-causing?)	Tolerated	C0	Polymorphism (*p* = 0.964)	None	1^a^
c.4964C>T	p.(Ser1655Phe)	16	Missense	rs80357390	RCV000112436.1: VUS RCV000223580.1: Likely pathogenic			CM041700(Disease-causing)	Deleterious	C25	Disease-causing (*p* = 1)	None	4^b^
c.5075A>C	p.(Asp1692Ala)	18	Missense	rs397509222	RCV000500821.1: VUS RCV000241473.1: VUS			CM169296(Disease-causing)	Deleterious	C65	Disease-causing (*p* = 1)	None	4
c.5095C>T	p.(Arg1699Trp)	18	Missense	rs55770810	RCV000048789.10: Pathogenic RCV000159999.4: Pathogenic RCV000077595.6: Pathogenic (ENIGMA) RCV000191041.1: Pathogenic RCV000457515.1: Pathogenic RCV000239322.2: Pathogenic RCV000131821.4: PathogenicRCV000148390.1: Pathogenic	ALL: 0.0024%EAS: 0.0058% NFE: 0.0018%FIN: 0.0090%OTH: *T* = 0.11%	EA: 0.01%	CM041706(Disease-causing)	Deleterious	C65	Disease-causing (*p* = 1)	None	5^b^
c.5096G>A	p.(Arg1699Gln)	18	Missense	rs41293459	RCV000031217.14: Likely pathogenic RCV000048790.6: Pathogenic/Likely pathogenic RCV000195350.6: Pathogenic/Likely pathogenicRCV000131564.6: Pathogenic/Likely pathogenic	ALL: 0.0024%NFE: 0.0054%		CM034007(Disease-causing)	Deleterious	C35	Disease-causing (*p* = 1)	None	4
c.5100A>G	p.(Thr1700Thr)	18	Synonymous	rs45519437	RCV000199783.5: Likely benign RCV000428938.2: Benign/Likely benign RCV000494789.2: Likely benign (ENIGMA) RCV000163399.2: Likely benign	ALL: 0.0028%AMR: 0.0060%SAS: 0.0032%NFE: 0.0036%	EA: 0.01%					None	2
c.5116G>A	p.(Gly1706Arg)	18	Missense	rs886040864	RCV000494689.1: Pathogenic RCV000257990.3: Likely pathogenic/VUS			CM1612904(Disease-causing?)	Deleterious	C65	Disease-causing (*p* = 1)	None	4
c.5123C>T	p.(Ala1708Val)	18	Missense	rs28897696	RCV000212194.4: VUS RCV000148393.1: VUS RCV000031221.5: VUS RCV000131166.5: VUS RCV000048803.10: VUS	ALL: 0.0024% AFR: 0.039%	EA: 0.01%AA: 0.05%	CM065004(Disease-causing)	Deleterious	C65	Disease-causing (*p* = 1)	None	3
c.5125G>A	p.(Gly1709Arg)	18	Missense	rs886038197	RCV000546570.2: VUS RCV000241163.1: VUS RCV000571176.2: VUS				Deleterious	C15	Disease-causing (*p* = 1)	None	3
c.5131A>C	p.(Lys1711Gln)	18	Missense		RCV000463327.1: VUS				Tolerated	C0	Disease-causing (*p* = 0.974)	None	3
c.5252G>A	p.(Arg1751Gln)	20	Missense	rs80357442	RCV000112579.2: Benign (ENIGMA) RCV000257892.6: Benign RCV000162992.3: Benign/Likely benign RCV000168520.7: Benign/Likely benign/VUS RCV000148392.1: VUS	ALL: 0.0041% AMR: 0.0060% NFE: 0.0072%	EA: 0.01%	CM022328(Disease-causing?)	Deleterious	C0	Disease-causing (*p* = 0.999)	None	1^a^
c.5309G>T	p.(Gly1770Val)	21	Missense		RCV000502156.1: Likely pathogenic RCV000477771.1: Likely pathogenic			CM133533(Disease-causing)	Deleterious	C0	Disease-causing (*p* = 1)	None	4^b^
c.5326C>T	p.(Pro1776Ser)	21	Missense	rs1800757	RCV000480229.1: VUS RCV000477350.2: VUS		EA: 0.01%		Tolerated	C0	Polymorphism (*p* = 0.741)	None	2
c.5348T>C	p.(Met1783Thr)	22	Missense	rs55808233	RCV000048954.7: Likely benign RCV000414204.1: VUS RCV000129758.4: Benign/Likely benign RCV000167822.8: Benign/Likely benign/VUS RCV000031240.7: Likely benign	ALL: 0.012%AFR: 0.18%AMR: 0.0060% OTH: 0.018%	AA: 0.18%	CM041721(Disease-causing?)	Deleterious	C45	Disease-causing (*p* = 0.999)	None	2
c.5411T>A	p.(Val1804Asp)	23	Missense	rs80356920	RCV000167770.7: Benign RCV000162993.3: Benign/Likely benign RCV000120302.4: Likely benign RCV000148405.1: VUS RCV000112647.4: Benign (ENIGMA)	ALL: 0.010% AMR: 0.048%NFE: 0.0027%	EA: 0.02%	CM044859(Disease-causing?)	Tolerated	C0	Polymorphism (*p* = 1)	None	2^a^
c.5477A>T	p.(Glu1826Leu)	24	Missense	rs730881499	RCV000160011.1: VUS	ALL: 0.0033% NFE: 0.0018% FIN: 0.027%			Tolerated	C0	Polymorphism (*p* = 0.763)	None	2
c.5504G>A	p.(Arg1835Gln)	24	Missense	rs273902776	RCV000049023.6: VUS RCV000240743.1: VUS RCV000130437.5: VUS RCV000112685.1: VUS RCV000120265.3: VUS	ALL: 0.0028% AFR: 0.013% EAS: 0.0058% NFE: 0.00090%			Tolerated	C0	Disease-causing (*p* = 0.965)	None	3
c.5513T>G	p.(Val1838Gly)	24	Missense	rs80357107	RCV000241502.1: Likely pathogenic			CM169297(Disease-causing)	Deleterious	C35	Disease-causing (*p* = 1)	None	4

Variant predictions by SIFT, AlignGVGD and Mutation taster, as well as splicing effects predicted by SpliceSiteFinder-like, MaxEntScan, NNSPLICE and GeneSplicer. Alterations of ≥ 10% and agreement between three or more splice software were used as criteria for a variant to likely result in aberrant splicing. Class is the final classification following the ACMG 5-tier scheme, combining TA assay results and available data. Class with indications for benign (^a^) and pathogenic (^b^) were used as controls and were classified according to the ACMG criteria prior to this study

### In silico assessment of *BRCA1* variants

All variants included in this study were analysed in silico using Alamut Visual 2.9.0. Table 1 displays entries in databases dbSNP, ClinVar, and HGMD, allele frequencies reported by gnomAD and ESP, where available, as well as the predicted effects of the variants based on reports from SIFT, AlignGVGD and Mutation taster. The maximal pathogenic allele frequency (MPAF) for *BRCA1* is estimated at 0.1%, variant allele frequencies above the MPAF (> 0.1) were considered evidence for the variant to be benign [25]. Splice predictions were based on SpliceSiteFinder-like, MaxEntScan, NNSPLICE and GeneSplicer, alterations of ≥ 10% and agreement between three or more programs were used as criteria for a variant to likely result in aberrant splicing. Variant effects on splicing regulatory elements (SREs) were not included in the predictions.

### Plasmid preparation and mutagenesis

The *BRCA1* BRCT variants were introduced into the pcDNA3 GAL4 DBD:BRCA1(aa 1396–1863) plasmid (kindly provided by Alvaro N. A. Monteiro) with the QuikChange II XL Site-Directed Mutagenesis Kit procedure (Agilent Technologies, Santa Clara, CA, USA), as per manufacturer’s instructions. Primers used for mutagenesis are summarised in Additional file 1: Table S1. Purification of plasmids was performed using the ZymoPURE™ Plasmid Maxiprep Kit and Zyppy™ Plasmid Miniprep Kit (Zymo Research, Irvine, CA, USA). Miniprep and the subsequent Maxiprep was performed once for each plasmid and used for all downstream applications. Plasmid quantification was performed using the Nanodrop® ND1000, and a 260/280 ratio between 1.7 and 1.9 was deemed satisfactory for plasmid purity. The quality of the plasmids was checked on an agarose gel. Correct incorporation of variants into pcDNA3 GAL4 DBD:BRCA1(aa 1396–1863) plasmid was verified using Sanger sequencing and the BigDye® Terminator v3.1. Cycle Sequencing Kit (Thermo Fisher, Waltham, MA, USA).

### Transfection and cell cultivation

HEK293T (ATCC® CRL-3216™) and MDA-MB-231 (ATCC® HTB-26™) cells were cultured in Dulbecco’s modified Eagle medium (DMEM) supplemented with 10% foetal bovine serum (FBS) and kept below 90% confluency prior to transfection experiments. The Lipofectamine® 3000 Transfection Reagent Kit (Invitrogen, Thermo Fisher, Waltham, MA, USA) was used for reverse-co-transfection of 0.2 μg reporter plasmid pGAL4-e1b-Luc (Firefly luciferase), 0.2 μg of pcDNA3 GAL4 DBD:BRCA1(aa 1396–1863) plasmid and 20 ng of reporter plasmid phRG-TK (Renilla luciferase). Ratios of Lipofectamine 3000 to P3000 were 1.75:1 for HEK293T and 1.2:1 for the MDA-MB-231. Cell suspensions of 4.0 × 10^4^ HEK293T or MDA-MB-231 cells were added to the transfection mix in 96-well plates. Cells transfected exclusively with reporter plasmids pGAL4-e1b-Luc and phRG-TK were used to measure background. Cells transfected with pcDNA3 GAL4 DBD:BRCA1(aa 1396–1863) containing *BRCA1*(aa 1396–1863) wild type (wt) sequence, benign/likely benign variants c.4956G>A, c.5252G>A and c.5411T>A or pathogenic/likely pathogenic variants c.4964C>T, c.5095C>T and c.5309G>T, were used as controls. Cells were harvested 24 and 48 h post-transfection. All experiments were performed in sextuplicates and repeated at least three times.

### Luciferase measurement

Luciferase measurements were conducted using the Dual Luciferase® Reporter Assay System Kit (Promega, Madison, Wi, USA) according to manufacturer’s instructions. Using a BioTek® Synergy H1 luminometer (BioTek, Winooski, VT, USA); 50 μL of Luciferase Assay Reagent II (LARII) was injected into 5 μL cell lysate in a white half area μclear® 96-well plate (Greiner bio-one, Monroe, NC, USA) followed by light emission measurement and subsequent injection of 50 μL Stop&Glo reagent and final measurement. The Firefly/Renilla ratio was used to mitigate possible differences in transfection efficiencies and cell numbers. The mean of each sextuplicate was calculated and presented as the percentage of wt pcDNA3 GAL4 DBD:BRCA1(aa 1396–1863) activity. Student *T* test was used for statistical analysis, and *p* values < 0.05 was considered significant.

### Western blot

The transfection experiments described under *Transfection and cell cultivation* were scaled to a 12-well plate setup for expression analysis of the GAL4 DBD:BRCA1(aa 1396–1863) fusion protein constructs by western blot analysis. Ten microgramme of sample protein was run on a 10% Mini-PROTEAN® TGX™ gel (Bio-Rad Laboratories, Hercules, CA, USA) and blotted onto 0.2 μm Nitrocellulose Membranes (Bio-Rad Laboratories, Hercules, CA, USA). The membranes were blocked with 5% BSA. Primary immunoblot staining was done using BRCA1 (D-9):sc-6954 (Santa Cruz Biotechnology, Dallas, TX, USA) in a 1:1000 dilution, overnight at 4 °C, and followed by staining with secondary antibody m-IgGκ BP-HRP: sc-516102 (Santa Cruz Biotechnology, Dallas, TX, USA) (1:1000) for 1 h at room temperature, before exposure using the ECL™ Prime Western Blotting Detection Reagent (GE Healthcare, Buckinghamshire, UK) with ImageQuant™ LAS 4000 and ImageQuant™ TL 1D v8.1 (GE Healthcare, Buckinghamshire, UK).

### cDNA synthesis and mRNA expression

The cDNA synthesis was performed on equal amounts of RNA isolated from a 12-well transfection experiment, using the High Capacity cDNA Reverse Transcription kit (Applied Biosystems®, Thermo Fisher, Waltham, MA, USA) in accordance with manufacturer’s instructions. Relative expression was measured with Applied Biosystems™ QuantStudio™ 12K Flex Real-Time System using SYBR™ green (Thermo Fisher, Waltham, MA, USA), and with *GAPDH* as reference gene. Relative expression (RQ) of pcDNA3 GAL4 DBD:BRCA1(aa 1396–1863) was calculated utilising the comparative ΔCt method with *GAPDH* as reference gene. Since amplification of pcDNA3 GAL4 DBD:BRCA1(aa 1396–1863) using SYBR™ green and *BRCA-*specific primers (Additional file 1: Table S2) also targets expression of endogenous *BRCA1*, non-transfected cells were used as reference to account for this.

## Results

### Trans-activation activity

**Fig. 2:**
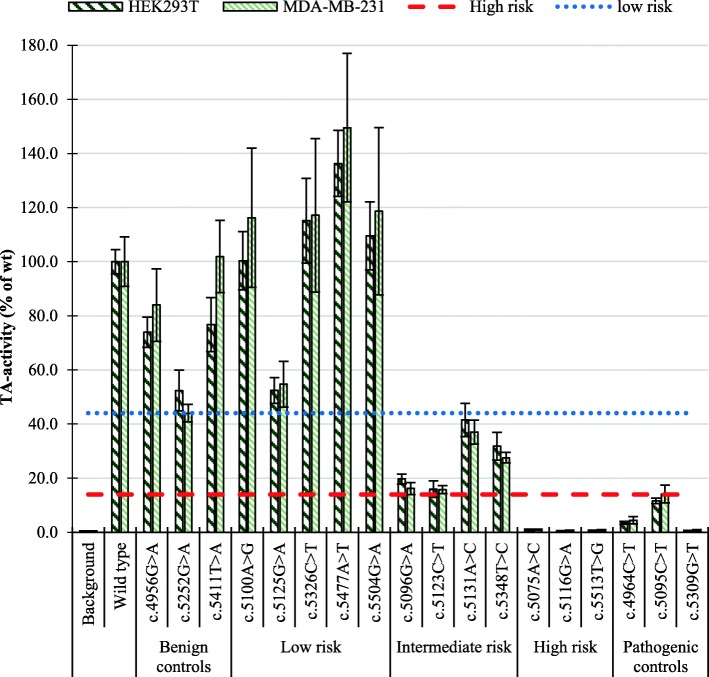
TA activity of *BRCA1* BRCT variants in HEK293T (dark green), MDA-MB-231 (light green) cells. Activities were measured after 48-h post-transfection incubation. The area below the lower (red) line indicates high risk, the area above the upper (blue) line indicates low risk. The area between these lines identifies variants of intermediate risk. TA activity is displayed as mean percentage of wt for three to four experiments conducted in sextuplicates, with error bars representing the standard deviation (*n* ≥ 18). The background is measured in cells transfected exclusively with reporter plasmids pGAL4-e1b-Luc (Firefly) and phRG-TK (Renilla)

Studying the effect of the *BRCA1* BRCT variants on the TA activity was performed in HEK293T and MDA-MB-231 cells after 48-h post-transfection incubation, for all variants included. The results (Fig. 2) revealed a high degree of TA activity similarity between the two cell lines. Variants with prior classification as likely benign/benign or likely pathogenic/pathogenic were used to estimate the range of TA activity in benign and deleterious variants, respectively. The 12 variants investigated in this study were divided into three groups based on a strict interpretation of the TA assay controls; no or low risk (TA ≥ 44%), high risk (TA ≤ 14%) and intermediate (14% < TA < 44%) (Fig. 2). Variants c.5096G>A and c.5123C>T displayed TA activities slightly above the estimated 14% threshold for pathogenicity (16.1–19.9% and 15.7–16.0% in MDA-MB-231 and HEK293T, respectively), but were not significantly different from the pathogenic control in the MDA-MB-231 cell line (*p* values 0.12 and 0.18 for c.5096G>A and c.5123C>T, respectively).

**Fig. 3:**
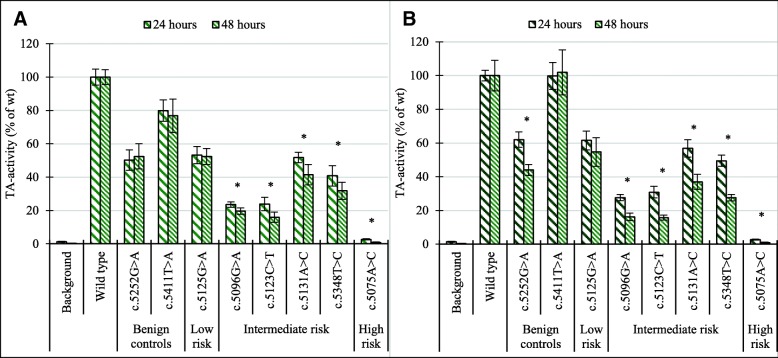
Effect of post-transfection incubation times on TA assay sensitivity. Comparison of the TA activity for selected variants after 24- (dark green) and 48 (light green)-h post-transfection incubation time. **a** HEK293T cells. **b** MDA-MB-231. TA activity is displayed as the percentage of wt trans-activation activity. Error bars represent standard deviations (*n* ≥ 18) for three to four experiments (*p value < 0.001)

The effect of prolonged post-transfection incubation time on the sensitivity of the TA assay yielded a significant increase for variants with TA activities < 50% after 48-h incubation, compared to 24-h in both HEK293T and MDA-MB-231 cells (*p* values < 0.001, Fig. 3a, b). The difference in TA activity between variants c.5131A>C and c.5348T>C (Fig. 3b) illustrated the increased sensitivity after 48 h by displaying a variation in TA activities that were not as clearly evident after 24 h.

### Additive effects of variants

**Fig. 4:**
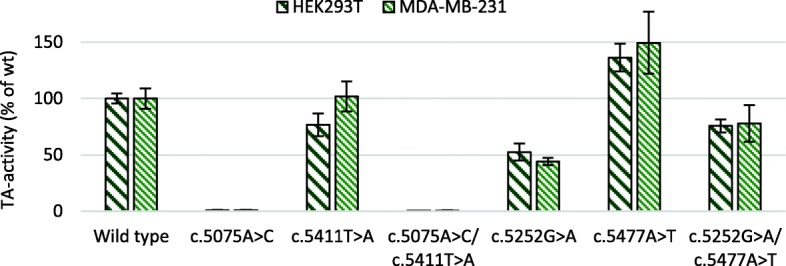
Additive effect of *BRCA1* BRCT variants *in cis*. TA activity as mean percentage of wt for plasmids containing the *in cis* variants c.5075A>C/c.5411T>A and c.5252G>A/c.5477A>T was analysed using HEK293T and MDA-MB-231 cells with 48-h post-transfection incubation time and three to four experiments in sextuplicates. Error bars represent standard deviation (*n* ≥ 18)

The TA activity of the plasmids containing the *in cis* variant combinations c.5075A>C/c.5411T>A and c.5252G>A/c.5477A>T were significantly different compared to the activity of plasmids containing each of these variants separately (Fig. 4, *p* values < 0.0001). The TA activity of c.5075A>C was low to begin with (0.86% in HEK293T and 0.91% in MDA-MB-231), inclusion of c.5411T>A reduced the TA activities additionally to 0.48% in HEK293T and 0.61% in MDA-MB-231. Variant c.5252G>A had TA activities of 52 and 44% of wt activity in cell lines HEK293T and MDA-MB-231, respectively, while variant c.5477A>T displayed increased TA activities in both cell lines; 136% in HEK293T and 150% in MDA-MB-231. The plasmid containing both variants had a TA activity of 76% in HEK293T and 78% in MDA-MB-231, and c.5477A>T seemed to rescue some of the loss in TA activity caused by c.5252G>A.

### Expression of the GAL4 DBD:BRCA1(aa 1396–1863) fusion protein

To confirm that the lack of TA activity was a result of the variant in question and not an inability to express the variant fusion protein, western blot analysis was performed on RIPA lysates from transfected HEK293T and MDA-MB-231 cells 48 h post-transfection (Additional file 1: Figure S1A and B, and Table S3). Cells transfected exclusively with the reporter plasmids and non-transfected cells were used as controls. Bands specific for the GAL4 DBD:BRCA1(aa 1396–1863) fusion protein were detected for all tested variants. Variants lacking TA activity (c.5075A>C and c.5309G>T) displayed weaker bands compared to the wt in HEK293T, but intensities comparable to wt in MDA-MB-231. Whereas variant c.5326C>T (TA activity > 100% in both cell lines) displayed weaker band intensity in MDA-MB-231 lysates than wt. Variant c.4964C>T, a positive control, with TA activity of 3.5% displayed band intensity comparable to the wt in HEK293T, and was stronger than the band observed for c.5348T>C with 32% TA activity, indicating a lack of correlation between TA activity and band intensity.

### mRNA expression of the pcDNA3 GAL4 DBD:BRCA1(aa 1396–1863) fusion protein

**Fig. 5:**
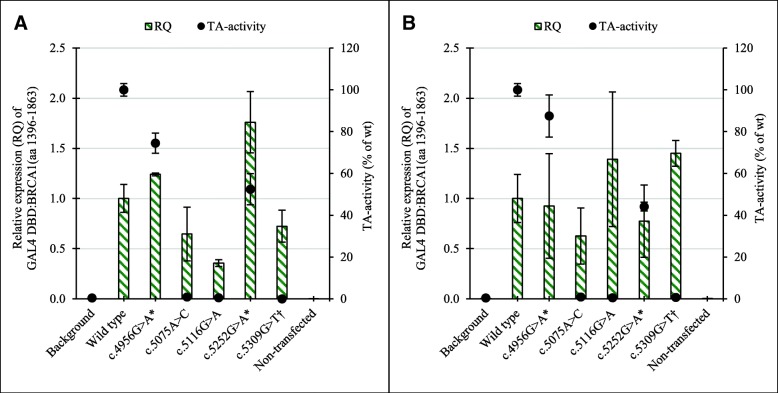
Mean relative expression (RQ) of variant GAL4 DBD:BRCA1(aa 1396–1863) fusion protein in transfected cell lines. **a** HEK293T and **b** MDA-MB-231 (green bars) with corresponding TA activities (black dots). RQ is displayed as the relative expression between pcDNA3 GAL4 DBD:BRCA1(aa 1396–1863) and GAPDH. Error bars represent standard deviations (*n*_RQ_ = 6, *n*_TA_ ≤ 18). The graph illustrates the independence between RQ and TA activity and is based on three qPCR experiments and three to four TA assay experiments

The pcDNA3 GAL4 DBD:BRCA1(aa 1396–1863) mRNA expression results provided a control of the TA assay in addition to the western blots. The variability observed in pcDNA3 GAL4 DBD:BRCA1(aa 1396–1863) mRNA expression between wt, variants and cell lines (Fig. 5a, b) does not translate to the TA activities. Measured TA activity remains highly similar between cell lines despite variations in the observed mRNA levels. There seem, however, to be a correlation between the western blot band intensities and the mRNA expression levels. Our results suggest that the measured TA activities were independent of mRNA levels and that they represent functional variant effects on the protein level.

## Discussion

### Risk thresholds

In order to use the TA assay for dividing variants into high- or low-risk categories, it was useful to define thresholds for measured TA activities representing a high- or low-risk variant. In a study by Carvalho et al., TA activity thresholds of ≤ 45% and ≥ 50% were proposed for categorising a variant as *high risk* or *low risk*, respectively [26]. While these thresholds likely remain true for the Carvalho et al.’s version of the TA assay, it is not apparent that this is a universal threshold that can be readily applied to all versions of the assay. Variant c.5095C>T (class 5) was used as a pathogenic control in our version of the TA assay and presented TA activities of 12 and 14% in HEK293T and MDA-MB-231 cells, respectively. This is a significant reduction in TA activity compared to the 45% reported by Carvalho et al. for the same variant and could be explained by the increased sensitivity of the assay presented here. The benign class 1 and 2 controls (c.4956G>A, c.5252G>A and c.5411T>A) were used to define the wt TA activity range, with the lowest observed TA activity in variant c.5252G>A, displaying TA activities of 52 and 44% in HEK293T and MDA-MB-231 cells, respectively. The threshold for what ought to be regarded as high risk in this version of the TA assay was set at ≤ 14%, and the low-risk threshold at ≥ 44%. As no gain-of-function regarding TA activity has been reported to confer with pathogenicity, no upper wt boundary was set.

### Risk assessment of *BRCA1* variants

The variants c.5100A>G, c.5125G>A, c.5326C>T, c.5477A>T and c.5504G>A presented with TA activities in the likely no/low-risk range defined by the TA assay controls and were therefore placed in the low-risk category. Four of these variants (c.5100A>G, c.5326C>T, c.5477A>T and c.5504G>A) presented TA activities higher than wt, whereas variant c.5125G>A displayed TA activity at the lower end of the low-risk range. To our knowledge, no functional assays have been performed on these variants except for c.5125G>A and c.5504G>A. Variant c.5125G>A was reported to display TA activity similar to wt, and c.5504G>A was found to have reduced homologous DNA recombination (HDR) capabilities compared to wt. [27, 28]. A computational method has also been utilised to predict the impact of variant c.5504G>A, but no evidence for a damaging effect was reported [29]. Our TA assay results in combination with the available data support a benign interpretation of variants c.5100A>G, c.5326C>T and c.5477A>T and that they are likely benign (class 2). Variant c.5125G>A could be considered a benign variant based on the TA assay results. However, due to in silico predictions of a deleterious nature and the lack of alleles in a control population, the classification of this variant should be further investigated to rule out any uncertainty and therefore retains its status as a VUS (class 3). Variant c.5504G>A remains classified as a VUS despite wt-like results on the TA assay due to conflicting reports of a possibly deleterious impact on HDR functionality.

Variants c.5075A>C, c.5116G>A and c.5513T>G displayed a complete lack of TA activity on our TA assay, consistent with pathogenic variant behaviour and previously reported TA results for c.5075A>C and c.5513T>G [27]. The three variants were predicted to have a deleterious nature by all applied software, and none were reported in control populations, further supporting a deleterious interpretation. The variants were known to ClinVar where variants c.5075A>C and c.5513T>G were reported as VUSs, while c.5116G>A was reported as disease-causing. Variants c.5075A>C and c.5513T>G have been reported previously to abolish TA activity [27], and to our knowledge, no other functional assays have been performed on these variants prior to this study. Based on these data, it seems likely that all three variants represent likely pathogenic (class 4) *BRCA1* variants.

Variants c.5096G>A, c.5123C>T, c.5131A>C and c.5348T>C presented TA activities in the intermediate range on the TA assay, and therefore, any risk related to these variants could not be ascertained based on the TA assay alone. However, variants c.5096G>A and c.5123C>T failed to present a significant difference in TA activity compared to pathogenic control c.5095C>T in the MDA-MB-231 cell line. Variant c.5096G>A has been reported to possess a deleterious effect on BRCA1 in multiple functional assays [30–33], with in silico analysis predicting a deleterious effect. However, the variant has been shown to result in a lower risk of cancer development than what is typically observed for *BRCA1* variants [33, 34]. Variant c.5123C>T has been reported to be functionally compromised in multiple functional assays [30] and was suggested to be a moderately penetrant variant in a study using a multifactorial likelihood analysis and multiple functional assays [35]. The status of variant c.5096G>A as a likely pathogenic variant with moderate penetrance is rather well documented, and while c.5123C>T may prove to be of a similar nature, we believe it should retain its status as a VUS until more data can be acquired.

Unlike the aforementioned intermediate variants, c.5131A>C displayed TA activities closer to the low-risk threshold. Analysis using in silico prediction software failed to agree on the nature of the variant, and it was absent in control populations, supporting a deleterious interpretation. Until a better assessment of c.5131A>C can be performed, it should likely remain classified as a VUS (class 3).

Variant c.5348T>C presented TA activities in the middle of the intermediate range established by the TA assay controls. In silico analysis of the variant predicted a deleterious effect on the BRCA1 protein, in all applied software. The influence of the variant on the function of the protein has been illustrated in some functional assays, whereas others have shown the variant to be either wt or inconclusive [30, 36–38]. The variant is known to ClinVar, with three reports of a benign nature, three reports of a likely benign nature and one report of unknown significance. The allele frequencies reported for the c.5348T>C variant were higher than what was expected of a pathogenic variant (Table 1) and has largely been found in an African and Afro-American population [39]. It is uncertain if the variant represents a benign variant with a lower TA activity than defined in this study or a potential risk factor. However, given the amount of evidence indicating a benign nature, c.5348T>C could be regarded as a likely benign, class 2, variant.

Recent advances in functional studies of *BRCA1* variants allow for a high throughput assessment of virtually any single nucleotide variant in the gene, or in functional domains of particular interest [40–42]. These methods have the distinct advantage of providing functional data on variants prior to their discovery in patients, thereby providing efficient classification relevant to clinical treatment. Comparing our results with those of Findlay et al. [40], we observe a high concordance between the TA assay and the saturation genome editing (SGE) data, particularly in the TA assay groups of high and low risk, including the pathogenic and benign controls. The only variant in the high-/low-risk groups that was classified differently between the two studies was c.5504G>A, that presented with a low-risk TA result but scored as an intermediate risk variant in the SGE dataset. The highest variability between the studies was found in variants placed in the intermediate risk category on the TA assay; variants c.5123C>T, c.5131A>C and c.5348T>C scored as functional on the SGE assay (c.5096G>A was not evaluated in the SGE assay).

### Additive variant effects

To our knowledge, no publications have investigated the effects of *BRCA1* variants *in cis* prior to our work, except for an investigation into the possible effects of including a polymorphism in combination with deleterious variants and VUSs that were unable to find any significant impact on TA activities [26]. We found that both *in cis* variant combinations tested revealed a significant effect. The combined variants c.5075A>C/c.5411T>A displayed an additional reduction in the TA activities compared to c.5075A>C alone; this could indicate that neutral variants can affect the performance of deleterious variants on the TA assay. Interestingly, the combination of c.5252G>A/c.5477A>T displayed TA activities in-between c.5252G>A and c.5477A>T alone, implying that the elevated TA activity of c.5477A>T was able to rescue some of the loss in the TA activity displayed by c.5252G>A. Considering the rarity of these particular variants, it seems unlikely that they will co-occur in any significant number of patients. However, it is an interesting observation that the co-occurrence of variants seemingly influences the performance of the TA assay in a synergistic manner and can potentially act as risk modifiers.

### Increased post-transfection incubation time

The effect of increased post-transfection incubation time on the sensitivity of the TA assay revealed a significant benefit using 48-h incubation instead of 24 h on variants with TA activity < 50%. Other attempts at investigating *BRCA1* variants utilising TA assays have usually been conducted with a post-transfection incubation period of 24 h [27, 30, 31, 43–45]. The improved sensitivity of the assay should enable a more precise distinction of intermediate variants that could be of clinical importance.

### Reproducibility of TA activities in different cell lines

The data obtained from the TA assays revealed little variability between cell lines, especially at low TA activities. The MDA-MB-231 cells generally displayed larger variability in the data than the HEK293T cells, especially in variants with TA activity > 70%. We conclude that the reproducibility of the TA assay results was high and that variant risk assessment did not differ between the cell lines.

### Limitations

There are several limitations to the TA assay, mainly that investigations of *BRCA1* variants utilising the TA assay are limited to variants in or near the BRCT domains. Second, the assay is performed only on a subsection of the protein, and how well the TA assay reflects variant effect on full-length BRCA1 is largely unknown. Additionally, a wt-like result on the TA assay cannot be regarded as conclusive evidence towards harmlessness, as the biological effect of the missense variant can escape detection on this assay (e.g. variants resulting in aberrant splicing). Despite this, the TA assay provides a reliable assessment of the BRCT-domain integrity, and the reported correlation between cancer predisposing variants and TA results is high [46]. The ability of the TA assay to assess the integrity of the BRCT domains makes it well suited for efficiently dividing *BRCA1* BRCT variants into risk groups but provides little or no explanation to the biological mechanism of how the variant contributes to tumorigenesis.

## Conclusion

In conclusion, the modified TA assay presented here provides efficient risk assessment of rare missense variants found in the BRCA1 BRCT domains. The increased post-transfection incubation time yielded a significant increase in TA assay sensitivity which may enable a better characterisation of BRCA1 BRCT variants with intermediate TA activity. The results presented here may aid in a better classification of the 12 included VUSs, a classification that is vital for the proper clinical care of affected patients.

## Additional file

Additional file 1: **Table S1.** Mutagenic primers used for introducing the variants into plasmid pcDNA3 GAL4 DBD:BRCA1(aa 1396–1863) by in vitro mutagenesis. The introduced variants are displayed in bold capital letters. Primers were designed according to the QuikChange II XL Site-Directed Mutagenesis Kit procedure (Agilent Technologies, Santa Clara, CA, USA) and provided by Eurofins (MWG Synthesis, GmbH). **Table S2.** Primers used for real-time quantification of endogenous *BRCA1* and plasmid pcDNA3 GAL4 DBD:BRCA1(aa 1396–1863). **Figure S1.** Western blot illustrating the presence of GAL4 DBD:BRCA1(aa 1396–1863) fusion protein in transfected cell lines A) HEK293T and B) MDA-MB-231 protein lysates from TA assay. Figure legends are shown in Additional file 1: **Table S2.** Variant c.5513T>G was only analysed on western blot in MDA-MB-231 cells. Band specific for GAL4 DBD:BRCA1(aa 1396–1863) (~ 80 kDa) and loading control β-actin (42 kDa) are indicated by black arrows. Ladder sizes 50 and 75 kDa are indicated. Blots represent one representative gel for each transfected cell line. **Table S3.** Western blot legend displaying the well number for each variant/sample and its corresponding TA activity for blots A and B (Figure S1a, b). Variants with indications were benign (*) and pathogenic (†) controls. (DOCX 376 kb)

## Abbreviations

aa: Amino acid
BRCT: BRCA1 C-terminal
DBD: DNA-binding domain
HBOC: Hereditary Breast and Ovarian Cancer
HRR: Homologous recombination repair
MPAF: Maximal pathogenic allele frequency
RQ: Relative quantification
SRE: Splicing regulatory element
TA: Trans-activation
wt: Wild type
