# Supplementary material for: Trans-activation-based risk assessment of BRCA1 BRCT variants with unknown clinical significance
Source: Hum Genomics. 2018 Nov 20;12:51. doi: 10.1186/s40246-018-0183-1 (PMC6247502; doi:10.1186/s40246-018-0183-1)
Supplement: Supplementary file 1 — Table S1. Mutagenic primers used for introducing the variants into plasmid pcDNA3 GAL4 DBD:BRCA1(aa 1396–1863) by in vitro mutagenesis. The introduced variants are displayed in bold capital letters. Primers were designed according to the QuikChange II XL Site-Directed Mutagenesis Kit procedure (Agilent Technologies, Santa Clara, CA, USA) and provided by Eurofins (MWG Synthesis, GmbH). Table S2. Primers used for real-time quantification of endogenous BRCA1 and plasmid pcDNA3 GAL4 DBD:BRCA1(aa 1396–1863). Figure S1. Western blot illustrating the presence of GAL4 DBD:BRCA1(aa 1396–1863) fusion protein in transfected cell lines A) HEK293T and B) MDA-MB-231 protein lysates from TA assay. Figure legends are shown in Additional file 1: Table S2. Variant c.5513T>G was only analysed on western blot in MDA-MB-231 cells. Band specific for GAL4 DBD:BRCA1(aa 1396–1863) (~ 80 kDa) and loading control β-actin (42 kDa) are indicated by black arrows. Ladder sizes 50 and 75 kDa are indicated. Blots represent one representative gel for each transfected cell line. Table S3. Western blot legend displaying the well number for each variant/sample and its corresponding TA activity for blots A and B (Figure S1a, b). Variants with indications were benign (*) and pathogenic (†) controls. (DOCX 376 kb) [file 40246_2018_183_MOESM1_ESM.docx]

**Supplementary information**

**Trans-activation based risk assessment of *BRCA1* BRCT variants with unknown clinical significance**

Jonas Langerud^1^, Elisabeth Jarhelle^2^, Marijke Van Ghelue^2^, Sarah Ariansen^1^, Nina Iversen^1*^

^1^ Department of Medical Genetics, Oslo University Hospital, Oslo, Norway

^2^ Department of Medical Genetics, Division of Child and Adolescent Health, University Hospital of North Norway, Tromsø, Norway

*Corresponding author:

Nina Iversen: [ninaiversenous@gmail.com](mailto:ninaiversenous@gmail.com)

Department of Medical Genetics, Oslo University Hospital, Oslo, Norway.
Ullevål sykehus
Postboks 4956 Nydalen
0424 Oslo

**Table S1.** Mutagenic primers used for introducing the variants into plasmid pcDNA3 GAL4 DBD:BRCA1(aa 1396-1863) by in vitro mutagenesis. The introduced variants are displayed in bold capital letters. Primers were designed according to the QuikChange II XL Site-Directed Mutagenesis Kit procedure (Agilent Technologies, Santa Clara, CA, USA), and provided by Eurofins (MWG Synthesis, GmbH).

| **HGVS nucleotide variant** | **Direction** | **Sequence 5'- 3'** |
| --- | --- | --- |
| c.4956G>A | fwd rev | ggggtcaggccagacaccac**T**atggacattcttttgttg caacaaaagaatgtccat**A**gtggtgtctggcctgacccc |
| c.4964C>T | fwd rev | ctggggtcaggcca**A**acaccaccatggacattc gaatgtccatggtggtgt**T**tggcctgaccccag |
| c.5075A>C | fwd rev | ccgttcacacacaaactcagca**G**ctgttttcataacaacatg catgttgttatgaaaacag**C**tgctgagtttgtgtgtgaacgg |
| c.5095C>T | fwd rev | cctagaaaatatttcagtgtcc**A**ttcacacacaaactcagc gctgagtttgtgtgtgaa**T**ggacactgaaatattttctagg |
| c.5096G>A | fwd rev | cctagaaaatatttcagtgtc**T**gttcacacacaaactcagc gctgagtttgtgtgtgaac**A**gacactgaaatattttctagg |
| c.5100A>G | fwd rev | cccgcaattcctagaaaatatttcag**C**gtccgttcacacacaaac gtttgtgtgtgaacggac**G**ctgaaatattttctaggaattgcggg |
| c.5116G>A | fwd rev | cccattttcctcccgcaattc**T**tagaaaatatttcagtg cactgaaatattttcta**A**gaattgcgggaggaaaatggg |
| c.5123C>T | fwd rev | ctacccattttcctccc**A**caattcctagaaaatatttcagtg cactgaaatattttctaggaattg**T**gggaggaaaatgggtag |
| c.5125G>A | fwd rev | ctacccattttcctc**T**cgcaattcctagaaaatatttcagtg cactgaaatattttctaggaattgcg**A**gaggaaaatgggtag |
| c.5131A>C | fwd rev | cccagaaatagctaactacccatt**G**tcctcccgcaattcctag ctaggaattgcgggagga**C**aatgggtagttagctatttctggg |
| c.5252G>A | fwd rev | ctgtcctgggattctcttgct**T**gctttggaccttggtggtttc gaaaccaccaaggtccaaagc**A**agcaagagaatcccaggacag |
| c.5309G>T | fwd rev | gtgggcatgttggtgaagggc**A**catagcaacagatttctag ctagaaatctgttgctatg**T**gcccttcaccaacatgcccac |
| c.5326C>T | fwd rev | ccattccagttgatctgtgg**A**catgttggtgaagggcccatagc gctatgggcccttcaccaacatg**T**ccacagatcaactggaatgg |
| c.5348T>C | fwd rev | gcaccacacagctgtacc**G**tccattccagttgatctgtgggc gcccacagatcaactggaatgga**C**ggtacagctgtgtggtgc |
| c.5411T>A | fwd rev | gcacaaccacaattgggtgg**T**cacctgtgccaagggtgaatg cattcacccttggcacaggtg**A**ccacccaattgtggttgtgc |
| c.5477A>T | fwd rev | caggtgcctcacacatc**A**gcccaattgcatggaagccattgtc gacaatggcttccatgcaattgggc**T**gatgtgtgaggcacctg |
| c.5504G>A | fwd rev | gctacactgtccaacacccactct**T**gggtcaccacaggtgcctc gaggcacctgtggtgaccc**A**agagtgggtgttggacagtgtagc |
| c.5513T>G | fwd rev | gtgctacactgtccaac**C**cccactctcgggtcaccac gtggtgacccgagagtggg**G**gttggacagtgtagcac |

**Table S2.** Primers used for real-time quantification of endogenous *BRCA1* and plasmid pcDNA3 GAL4 DBD:BRCA1(aa 1396-1863)*.*

| Name | Sequence **5'- 3'** |
| --- | --- |
| BRCA1_QPCR_14-15F | GAACCAGGAGTGGAAAGGTCAT |
| BRCA1_QPCR_15R2 | TGGGTAGTTTCTATTCTGAAGACT |


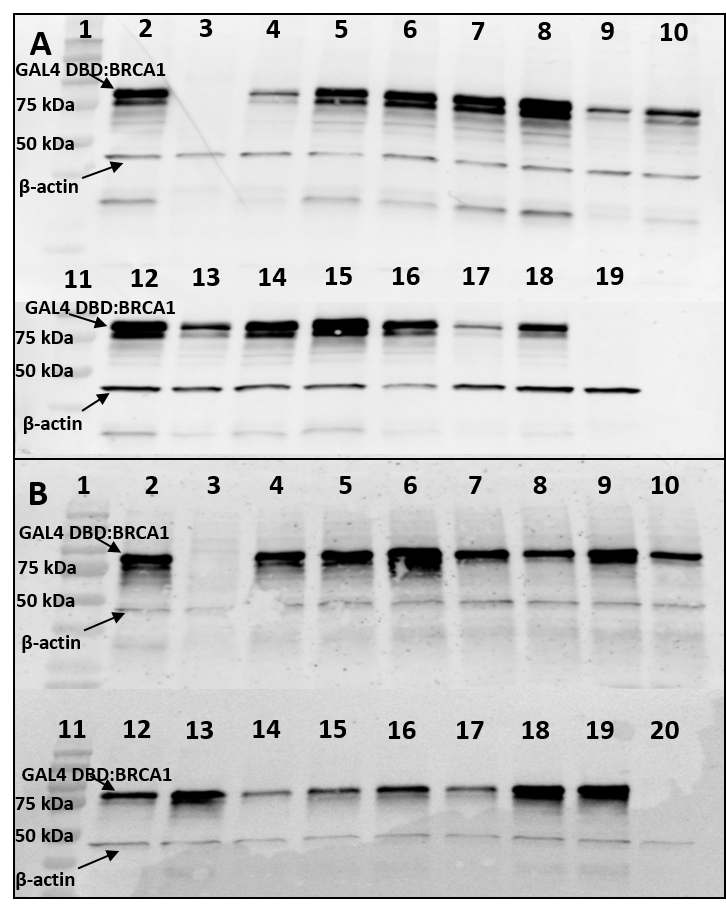


**Fig. S1 Western blot illustrating the presence of GAL4 DBD:BRCA1(aa1396‑1863) fusion protein in transfected cell lines** **A)** HEK293T and **B)** MDA-MB-231 protein lysates from TA-assay. Figure legend are shown in Supplementary Table 2. Variant c.5513T>G was only analysed on western blot in MDA-MB-231 cells. Band specific for GAL4 DBD:BRCA1(aa1396‑1863) (~80 kDa) and loading control β-actin (42 kDa) are indicated by black arrows. Ladder sizes 50 and 75 kDa are indicated. Blots represent one representative gel for each transfected cell line.

**Table S3.** Western blot legend displaying the well number for each variant/sample and its corresponding TA-activity for blots A and B (Fig. 6A and B). Variants with indications were benign (*) and pathogenic (†) controls.

| **Well** | **HGVS protein variant** | **TA-activity** | **Well** | **HGVS protein variant** | **TA-activity** |
| --- | --- | --- | --- | --- | --- |
| A-1 | Ladder | - | B-1 | Ladder | - |
| A-2 | Wild type | 100.0 % | B-2 | Wild type | 100.0 % |
| A-3 | Reporter | 0.5 % | B-3 | Reporter | 0.5 % |
| A-4 | c.5075A>C | 0.8 % | B-4 | c.4956G>A* | 87.5 % |
| A-5 | c.4956G>A* | 74.5 % | B-5 | c.5252G>A* | 44.1 % |
| A-6 | c.5252G>A* | 52.4% | B-6 | c.5411T>A* | 103.0 % |
| A-7 | c.5411T>A* | 77.3 % | B-7 | c.5075A>C | 0.9 % |
| A-8 | c.5100A>G | 101.6 % | B-8 | c.5513T>G | 0.5 % |
| A-9 | c.5116G>A | 0.5 % | B-9 | c.4964C>T† | 4.5 % |
| A-10 | c.5131A>C | 41.6 % | B-10 | c.5309G>T† | 0.7 % |
| A-11 | Ladder | - | B-11 | Ladder | - |
| A-12 | c.5326C>T | 115.4 % | B-12 | c.5095C>T† | 14.4 % |
| A-13 | c.5348T>C | 31.9 % | B-13 | c.5100A>G | 119.7 % |
| A-14 | c.5477A>T | 136.4 % | B-14 | c.5116G>A | 0.5 % |
| A-15 | c.5504G>A | 109.8 % | B-15 | c.5131A>C | 36.8 % |
| A-16 | c.4964C>T† | 3.5 % | B-16 | c.5326C>T | 133.7 % |
| A-17 | c.5309G>T† | 0.5 % | B-17 | c.5348T>C | 27.2 % |
| A-18 | c.5095C>T† | 11.7 % | B-18 | c.5477A>T | 155.1 % |
| A-19 | Non-transfected | - | B-19 | c.5504G>A | 122.0 % |
|  |  |  | B-20 | Non transfected | - |
